# Cardiometabolic risk profile and associated factors among adults with type 2 diabetes in a primary health care clinics in North Central Nigeria

**DOI:** 10.4102/phcfm.v18i1.5461

**Published:** 2026-08-28

**Authors:** Innocent N. Okonkwo, Maryanna Idoh, Ezra O. Agbo, Edwin N. Okafor

**Affiliations:** 1 Department of Medical Laboratory Science, Faculty of Health Sciences and Technology, University of Nigeria, Enugu, Nigeria; 2 Department of Chemical Pathology, Benue State Teaching Hospital, Benue, Nigeria; 3 Department of Chemical Pathology, Faculty of Basic Clinical Sciences, College of Medicine and Health Sciences, Baze University, Abuja, Nigeria; 4 Department of Medical Laboratory Sciences, Faculty of Health Sciences and Technology, College of Medicine, University of Nigeria, Enugu, Nigeria

**Keywords:** type 2 diabetes, cardiometabolic abnormalities, renal impairment, primary health care, Nigeria

## Abstract

**Background:**

Cardiometabolic abnormalities substantially increase morbidity among individuals with type 2 diabetes mellitus (T2DM), particularly in primary health care (PHC) settings. In Nigeria, evidence on comprehensive metabolic risk profiling beyond glycaemic control remains limited.

**Aim:**

To determine the cardiometabolic risk profile and identify factors associated with adverse cardiometabolic outcomes among adults with T2DM.

**Setting:**

The study took place at government-owned PHC facilities in Makurdi, Benue State, Nigeria.

**Methods:**

This facility-based analytical cross-sectional study systematically recruited 120 eligible adults aged ≥ 30 years with T2DM. Socio-demographic and clinical data were obtained using structured questionnaires and medical records. Laboratory analyses assessed glycaemic status, lipid profile and renal function. Renal impairment was determined using estimated glomerular filtration rate (eGFR) based on standard clinical thresholds. Cardiometabolic abnormality was defined as the presence of at least one abnormal component: poor glycaemic control, dyslipidaemia or renal impairment. Data were analysed using descriptive statistics and logistic regression.

**Results:**

The mean age was 56 ± 10.8 years, with females comprising 61.7%. The mean diabetes duration was 4 ± 3.2 years. Overall, 84.8% had at least one cardiometabolic abnormality. Poor glycaemic control, dyslipidaemia and renal impairment occurred in 65.8%, 65.0% and 29.2%, respectively. The mean cardiometabolic risk score was 1.87 ± 1.05. Longer diabetes duration independently predicted poor glycaemic control (*p* < 0.05).

**Conclusion:**

Adults with T2DM attending PHC clinic had a high burden of cardiometabolic abnormalities, highlighting the need for integrated diabetes management strategies.

**Contribution:**

The study provides context-specific evidence to support routine lipid and renal function assessment in resource-limited settings.

## Introduction

Type 2 diabetes mellitus (T2DM) is a chronic metabolic disorder characterised by persistent hyperglycaemia resulting from insulin resistance and progressive β-cell dysfunction.^1^ It is a leading contributor to cardiovascular disease, chronic kidney disease and premature mortality globally.^2^ The International Diabetes Federation estimates that diabetes prevalence will continue to rise disproportionately in low- and middle-income countries, with sub-Saharan Africa projected to experience one of the highest percentage increases by 2045.^1,3^

Nigeria bears a growing burden of T2DM, driven by rapid urbanisation, nutrition transition, physical inactivity and demographic changes.^4,5^ National estimates suggest increasing prevalence alongside significant rates of undiagnosed disease.^4^ Beyond hyperglycaemia, individuals with T2DM frequently demonstrate that cardiometabolic risk refers to the co-existence of interrelated metabolic and cardiovascular abnormalities, such as hypertension, dyslipidaemia, obesity and renal dysfunction. Reports have documented that these abnormalities often co-exist in individuals with metabolic diseases such as T2DM and contribute to morbidity and mortality.^6,7^

These cardiometabolic abnormalities including dyslipidaemia and renal impairment, substantially increase the risk of atherosclerotic cardiovascular disease and account for the majority of deaths among people living with diabetes.^2,8,9^

Primary health care (PHC) forms the foundation of equitable and accessible health systems.^9^ Strong primary care systems are associated with improved chronic disease outcomes, reduced hospitalisation and lower health costs.^10,11,12^ The renewed global commitment to PHC strengthening, reaffirmed in the Declaration of Astana, highlights integrated, people-centred care as central to achieving universal health coverage.^13^ The World Health Organization (WHO) has further emphasised the critical role of PHC in addressing non-communicable diseases, including diabetes.^14^ In many low-resource settings, diabetes management at the PHC level remains predominantly focused on symptomatic treatment and glycaemic monitoring, with limited systematic assessment of broader cardiometabolic risk factors.^15,16^ However, international guidelines recommend routine lipid profiling and renal function assessment for comprehensive risk stratification in T2DM.^17,18^ Integrated cardiometabolic screening enables early identification of individuals at high cardiovascular and renal risk and supports timely intervention.^19^

Evidence from sub-Saharan Africa demonstrates a high prevalence of dyslipidaemia among people with T2DM, frequently under-diagnosed and undertreated.^20,21^ Similarly, diabetic kidney disease is increasingly recognised as a major contributor to morbidity and mortality in African populations.^22,23^ Despite this, routine renal assessment is inconsistently implemented in many PHC settings because of resource limitations and service fragmentation.^24^ Most Nigerian studies examining cardiometabolic abnormalities among individuals with T2DM have been conducted in tertiary or specialist centres.^4,25,26^ Data from PHC settings, where a substantial proportion of patients receive routine chronic care, remain limited. This represents an important knowledge gap, particularly in North-Central Nigeria. This study aimed to determine the cardiometabolic risk profile and associated factors among adults with T2DM in PHC clinics in North Central Nigeria.

## Research methods and design

### Study design and setting

An analytical facility-based cross-sectional study was conducted between September 2025 and February 2026 in three selected PHC clinics in Makurdi, Benue State, North Central Nigeria. The PHCs are government-owned and operate under the Benue State Primary Healthcare Board, serving as first-contact facilities for a large proportion of residents, including adults with T2DM. They are distributed across urban and peri-urban communities and provide outpatient services such as screening, diagnosis, routine follow-up, health education and basic management of diabetes, with referral to secondary or tertiary facilities when necessary. Diagnostic capacity is limited to basic investigations. The selected PHCs were chosen because they collectively managed a substantial number of diabetic patients, making them suitable for assessing cardiometabolic risk profiles in a real-world primary care setting. Cross-sectional epidemiological studies are appropriate for estimating disease burden and risk factor distribution within defined populations.^27,28^

### Study population

The study population consisted of adults aged ≥ 30 years with previously diagnosed T2DM who were receiving routine clinical care at the selected PHC facilities. Adults aged > 30 years with clinically stable T2DM were included after providing informed consent. Patients with type 1 diabetes mellitus, acute illness or hospitalisation, pregnancy and conditions likely to independently influence cardiometabolic parameters (e.g. advanced malignancy or severe hepatic diseases), as well as those who declined consent, were excluded.

### Sample size determination and sampling technique

The sample size was determined using the single population formula for cross-sectional studies, assuming an expected prevalence of 50% for cardiometabolic abnormalities among adults with T2DM, a 95% confidence level and a 9% margin of error. The prevalence of 50% was used as a conservative estimate, as no previous local prevalence data on cardiovascular abnormalities among selected adults with T2DM attending PHC clinics in Makurdi were available. Using these parameters, the minimum calculated sample size was approximately 119 participants, which was rounded up to 120 participants for the study. The sample size was considered adequate for facility-based descriptive prevalence estimation studies.^29^ Participants were selected using systematic sampling from clinic attendance registers to minimise selection bias and improve representativeness.^30^

### Data collection

#### Questionnaire development

The questionnaire was developed by adapting validated instruments and internationally recognised guidelines, including the WHO STEPwise approach and the American Diabetes Association Standards of Care.^31,32^ Core socio-demographic and clinical variables were retained, while the tool was modified to suit the PHC context by simplifying language and removing tertiary-care-specific items. Additional study-specific items were included to assess cardiometabolic investigations such as blood pressure monitoring, lipid profile and renal function tests. The instruments were reviewed by experts for content validity and pretested in a similar PHC setting prior to data collection.

Venous blood samples were collected after overnight fasting. Laboratory investigations included fasting blood glucose (FBG), glycated haemoglobin (HbA1c), lipid profile parameters (total cholesterol, low-density lipoprotein cholesterol, high-density lipoprotein cholesterol and triglycerides), and serum creatinine and urea, measured using standard laboratory procedures.

### Definition of outcome variables

Poor glycaemic control was defined as HbA1c ≥ 7% in accordance with international diabetes management guidelines.^17^ Renal impairment was defined as eGFR < 60 mL/min per 1.73 m.^33,34^ Estimated glomerular filtration rate (eGFR) was calculated using the 2021 chronic kidney disease-epidemiology collaboration (CKD-EPI) creatinine equation developed by Lesley A. Inker and colleagues. Because the study was cross-sectional, chronicity of renal dysfunction could not be confirmed; therefore, renal impairment was reported rather than chronic kidney disease. Dyslipidaemia was defined as total cholesterol ≥ 5.5 mmol/L, LDL-cholesterol ≥ 2.6 mmol/L, triglycerides ≥ 1.7 mmol/L and HDL-cholesterol < 1.0 mmol/L for males and < 1.3 mmol/L for females based on guideline recommendations of the WHO and American Diabetes Association. Cardiometabolic abnormalities were defined as the presence of one or more abnormal cardiometabolic components, including poor glycaemic control, dyslipidaemia or renal impairment determined using established guideline-recommended cut-off values.

### Quality control

Standard operating procedures were followed throughout the study. The questionnaire was pretested, and data were collected by trained personnel using calibrated equipment. Biological samples were collected, processed, and analysed according to established laboratory protocols with appropriate internal quality control measures. Data were checked for completeness, consistency and accuracy before analysis.

#### Validity and reliability of the data collection tool

The questionnaire was adapted from the WHO STEPwise approach to non-communicable disease risk factor surveillance and the American Diabetes Association Standards of Care. Face validity was assessed by experts in clinical biochemistry, public health and epidemiology to ensure that the items were clear, relevant and appropriate for the study objectives. Content validity was established through expert review to confirm that the questionnaire adequately covered the domains of sociodemographic characteristics, clinical history and cardiometabolic risk factors. Construct validity was ensured by aligning questionnaire items with established theoretical and empirical concepts underlying cardiometabolic risk assessment.

To ensure reliability, the questionnaire was pretested among adults with type 2 diabetes attending a PHC facility outside the study area. Feedback from the pretest was used to refine ambiguous items and improve clarity. Standardised data collection procedures were employed, and research assistants received training on questionnaire administration and measurement technique to ensure consistency and reproducibility of the data collected.

### Statistical analysis

Data were analysed using descriptive statistics including mean, standard deviation, frequencies and percentages. Chi-square test was used to assess bivariate associations. Multivariable logistic regression analysis was performed to determine independent predictors of poor glycaemic control. Statistical significance was set at *p* < 0.05.^35^

### Ethical considerations

Ethical approval for this study was obtained from the Health Research Ethics Committee (HREC) of Benue State University Teaching Hospital (BSUTH), Makurdi, Benue State, Nigeria. Approval number: BSUTH/MKD/HREC/2023/148. Permission was also obtained from relevant PHC authorities. Written informed consent was obtained. The study was conducted in accordance with the National Code of Health Research Ethics (Nigeria) and institutional ethical guidelines governing research involving human participants.^36^ Confidentiality was maintained by anonymising data, and all procedures adhered to the principles of the Declaration of Helsinki.

## Results

### Basic characteristics of participants

The baseline characteristics of all T2DM participants are listed in Table 1. A total of 120 adults with type 2 diabetes were included in the study. The majority of participants were aged 50–59 years (31.7%), followed by aged 40–49 years and 60–70 years with 26.7% and 26.6%, respectively, while 15% were aged 30–39 years. Females comprised a slightly higher proportion of the study population (61.7%) compared with males 38.3%. Regarding duration of diabetes, 33.3% had been diagnosed for 1–5 years, while 28.3% had diabetes for 6–10 years and 21.7% for ≥ 10 years; notably, 16.7% had been diagnosed for < 1 year. Most participants were managed with oral hypoglycaemic agents (61.7%), whereas 23.3% were on insulin therapy and 15.0% were receiving combination therapy.

**TABLE 1:** Baseline characteristics of the participants and average mean ± s.d. of demographics (*N* = 120).

Characteristic	Mean ± s.d.	*n*	%
Age (years)	56.4 ± 10.8	-	-
Duration of diabetes (years)	6.2 ± 4.1	-	-
Fasting plasma glucose (mmol/L)	9.8 ± 1.9	-	-
HbA1c (%)	8.4 ± 1.9	-	-
**Sex**			
Male	54.4 ± 8.0 years	46	38.3
Female	55.8 ± 7.5 years	74	61.7
**Age group (years)**			
30–39	-	18	15.0
40–49	-	32	26.7
50–59	-	38	31.7
60–70	-	32	26.6
**Duration categories (years)**			
< 1	-	20	16.7
1–5	-	40	33.3
6–10	-	34	28.3
≥ 10	-	26	21.7
**Treatment**			
Oral hypoglycaemic agents	-	74	61.7
Insulin therapy	-	28	23.3
Combination therapy	-	18	15.0
**Education completed**			
No formal school	-	25	20.8
Secondary school	-	50	41.7
Tertiary education	-	10	8.3
**Occupation**			
Unskilled	-	60	50.0
Skilled	-	20	16.7
Unemployed	-	30	25.0

### Glycaemic by duration

Participants with poor glycaemic control had higher mean HbA1c than those with good control (9.8 ± 1.8% vs 6.3 ± 0.5%; *p* < 0.001). Mean fasting plasma glucose was also significantly higher among those with poor control (9.8 ± 3.4 mmol/L) compared with participants with good control (6.4 ± 1.8 mmol/L; *p* < 0.001). The mean duration of diabetes was significantly longer in participants with poor glycaemic control (7.2 ± 4.8 years) than in those with good control (4.1 ± 3.2 years; *p* = 0.002). Of the 120 participants, 79 (65.8%) had poor glycaemic control, while 41 (34.2%) achieved good control (see Table 2 a–c).

**TABLE 2a:** Glyceamic parameters by glyceamic control status among adults with type 2 diabetes mellitus (*N* = 120).

Variable	Good control HbA1c < 7% (*n* = 41)	Poor control HbA1c ≥ 7% (*n* = 79)	*p*-value
HbA1c (%)	6.3 ± 0.5	9.8 ± 1.8	< 0.001*
Fasting plasma glucose (mmol/L)	6.4 ± 1.8	9.8 ± 3.4	< 0.001*
Duration of diabetes (years)	4.1 ± 3.2	7.2 ± 4.8	0.002*

Note: *p*-value from independent *t*-test for continuous variable. Renal impairment defined (eGFR < 60 mL/min per 1.73 m^2^).

HbA1c, glycated haemoglobin; mmol/L, millimole per litre.

* , Indicates a statistically significant difference between the groups (*p* < 0.05).

**TABLE 2b:** Glyceamic parameters by glyceamic control status among adults with type 2 diabetes mellitus (*N* = 120).

Category	Frequency (*n*)	%
Good glycaemic control	41	34.2
Poor glycaemic control	79	65.8

**TABLE 2c:** Glyceamic parameters by glyceamic control status among adults with type 2 diabetes mellitus (*N* = 120).

Duration category (year)	Good control (*n* = 41)		Poor control (*n* = 79)	
	*n*	%	*n*	%
< 1	7	17.1	8	10 0
1–5	12	29.3	16	20.3
6–10	10	24.3	30	38.0
≥ 10	12	29.3	25	31.7

### Lipid profile and dyslipidaemia

Participants with poor glycaemic control (*n* = 79) had a significantly more atherogenic lipid profile than those with good control (*n* = 41). Mean total cholesterol (5.5 ± 1.2 mmol/L), LDL cholesterol (3.6 ± 1.0 mmol/L) and triglyceride levels (2.2 ± 0.9 mmol/L) were significantly higher among participants with good control (4.6 ± 0.9 mmol/L, 2.6 ± 0.8 mmol/L and 1.5 ± 0.6 mmol/L, respectively, *p* < 0.001). Conversely, HDL-cholesterol was significantly lower in the poor control group (1.0 ± 0.2 mmol/L vs 1.3 ± 0.3 mmol/L, *p* = 0.01).

Overall, dyslipidaemia was present in 65% of participants and was significantly more common among those with poor glycaemic control (75.9% vs 43.9%, *p* = 0.001) (Table 3 a–c).

**TABLE 3a:** Glyceamic parameters by glyceamic control status among adults with type 2 diabetes mellitus (*N* = 120).

Lipid parameter	Good control (*n* = 41)	Poor control (*n* = 79)	*p*-value
Total cholesterol (mmol/L)	4.6 ± 0.9	5.5 ± 1.2	0.001*
LDL cholesterol (mmol/L)	2.6 ± 0.8	3.6 ± 1.0	< 0.001*
HDL cholesterol (mmol/L)	1.3 ± 0.3	1.0 ± 0.2	0.01*
Triglycerides (mmol/L)	1.5 ± 0.6	2.2 ± 0.9	0.001*

Note: Mean ± s.d.

LDL, low-density lipoprotein; HDL, high-density lipoprotein.

* , *p*-value < 0.05.

**TABLE 3b:** Overall prevalence of dyslipidaemia among adults with type 2 diabetes (*N* = 120).

Variable	Frequency (*n*)	%
Dyslipidaemia present	78	65.0
Normal lipid profile	42	35.0

Note: Number (%).

**TABLE 3c:** Association between glyceamic control status and dyslipidaemia among adults with type 2 diabetes mellitus (*N* = 120).

Glycaemic status	Dyslipidaemia		Normal lipid profile		*p*-value
	*n*	%	*n*	%	
Good control	18	43.9	23	36.1	-
Poor control	60	75.9	19	24.1	0.001*

Note: Number (%).

* , *p*-value < 0.05.

### Renal function and renal impairment

Participants with poor glycaemic control had significantly worse renal function compared with those with good control. Mean serum creatinine (108.45 ± 32.7 μmol/L vs 82.5 ± 18.6 μmol/L, *p* < 0.001 and mean blood urea (7.4 ± 2.5 mmol/L vs 5.2 ± 1.6 mmol/L, *p* = 0.001) were significantly higher among participants with poor glycaemic control. The mean eGFR of the participants was 74.6 ± 21.3 mL/min per 1.73 m^2^. Renal function assessed showed that 35 participants (29.2%) had renal impairment defined (eGFR < 60 mL/min per 1.73 m^2^), and 85 participants (70.8%) had preserved renal function (Table 4 a–c).

**TABLE 4a:** Renal function parameters by glyceamic control status among adults with type 2 diabetes (*N* = 120).

Renal parameter	Good control (*n* = 41)	Poor control (*n* = 79)	*p*-value
Serum creatinine (μmol/L)	82.5 ± 18.6	108.4 5 ± 32.7	0.001*
Blood urea (mmol/L)	5.2 5 ± 1.6	7.4 5 ± 2.5	0.001*

Note: Mean ± s.d.

μmmol/L, micromole per litre; mmol/L, millimole per litre.

* , *p*-value < 0.05.

**TABLE 4b:** Overall prevalence of renal impairment by glyceamic control status among adults with type 2 diabetes mellitus (*N* = 120).

Variable	Frequency (*n*)	%
Renal impairment present	35	29.2
Normal renal function	85	69.8

Note: Number (%).

* , *p*-value < 0.05.

**TABLE 4c:** Association between glyceamic control status and renal impairment among adults with type 2 diabetes mellitus (*N* = 120).

Glycaemic status	Renal impairment		Normal renal function		*p-value*
	*n*	%	*n*	%	
**Renal impairment vs glycaemic control**	-	-	-	-	0.01*
Good control	5	14.6	36.0	85.4	-
Poor control	30	38.8	49.0	62.2	-
**Renal function category**	-	-	-	-	-
≥ 60 (Normal to mildly decreased)	85	-	70.8	-	-
< 60 (Renal impairment)	35	-	29.2	-	-

**Total**	**120**	-	**100.0**	-	-

Note: Number (%).

* , *p*-value < 0.05.

### Distribution of cardiometabolic abnormalities

This shows the distribution of cardiometabolic abnormalities among the participants. Overall, 15.2% of the respondents had no cardiometabolic abnormality, while 16.4% had one abnormality. A substantial proportion of participants had multiple abnormalities, with 34.2% having two and another 34.2% having three abnormalities. The overall prevalence of cardiometabolic abnormalities (defined as the presence of at least one abnormal component) was 84.8%. The mean cardiometabolic risk score was 1.87 ± 1.05 (Table 5).

**TABLE 5:** Distribution and overall prevalence of cardiometabolic abnormalities among study population (*N* = 120).

Number of cardiometabolic abnormalities	Frequency (*n*)	%
None	18	15.2
One	20	16.4
Two	41	34.2
≥ Three	41	34.2
**Cardiometabolic abnormalities include poor glycaemic control, dyslipidaemia and renal impairment**		
Overall prevalence (≥ 1 abnormality)	-	84.8

Note: Mean cardiometabolic risk score: 1.87 ± 1.05. *p*-value chi-square < 0.05. Number (%).

Cardiometabolic abnormalities include poor glycaemic control, dyslipidaemia and renal impairment.

### Predictors of cardiometabolic clustering

Multivariable logistic regression analysis identified independent predictors of poor glycaemic control among the study participants. Longer duration of diabetes was significantly associated with increased odds of poor glycaemic control (adjusted odds ratio [AOR]: 1.12; 95% confidence interval [CI]: 1.03–1.22; *p* = 0.01), indicating that each additional year of diabetes increases the likelihood of poor control by 12%. Higher fasting plasma glucose was also independently associated with poor glycaemic control (AOR: 1.28; 95% CI: 1.10–1.48; *p* = 0.002). Participants with dyslipidaemia had nearly threefold odds of poor glycaemic control compared with those without dyslipidaemia (AOR: 2.84; 95% CI: 1.18–6.84; *p* = 0.02). Similarly, renal impairment was significantly associated with more than twofold increased odds of poor glycaemic control (AOR: 2.41; 95% CI: 1.01–5.76; *p* = 0.04). Age was not significantly associated with poor glycaemic control after adjustment (Table 6).

**TABLE 6:** Predictors of cardiometabolic clustering (*N* = 120).

Variable	Adjusted OR	95% CI	*p*-value
Age (years) (mean ± s.d.)	1.01	0.98–1.04	0.41
Duration of diabetes (years) (mean ± s.d.)	1.12	1.03–1.22	0.01
Fasting plasma glucose (mmol/L) (mean ± s.d.)	1.28	1.10–1.48	0.002
Dyslipidaemia	2.84	1.18–6.84	0.02
Renal impairment	2.14	1.01–6.76	0.04

Note: *p*-value < 0.05 (%). Mean ± s.d., adjusted 95% confidence interval.

s.d., standard deviation; CI, confidence interval; OR, odds ratio; mmol/L, millimole per litre.

## Discussion

This study demonstrated a high burden of cardiometabolic risk profile among adults with T2DM attending PHC clinics in North-Central Nigeria, with an overall prevalence of 84.8%. This finding highlights the substantial clustering of metabolic, cardiovascular, and renal abnormalities among individuals with T2DM. The high prevalence observed in the present study is consistent with the findings of Junaid et al., who reported a similarly high burden of cardiometabolic risk factors among Nigerian adults with T2DM, including dyslipidaemia (97.9%) and hypertension (77.1%).^37^ However, lower prevalence has been reported in other African populations. Owiredu et al. reported a prevalence of 24.0% among Ghanaian adults with T2DM,^38^ while Adediran et al. reported a prevalence of 51.0% among Nigerian patients with T2DM.^39^ This variation may be attributed to differences in study population, healthcare settings, duration of diabetes, diagnostic criteria, and the inclusion of renal abnormalities in the assessment of cardiometabolic risk. Nevertheless, the findings underscore the need for comprehensive cardiometabolic risk assessment and integrated management strategies among adults with T2DM, particularly within PHC settings.

The prevalence of poor glycaemic control observed in this study (65%) is comparable with recent findings from sub-Saharan Africa and other low- and middle-income countries. A recent prospective observational study reported poor glycaemic control in 58.1% of patients with type 2 diabetes, highlighting persistent challenges in achieving optimal glycaemic targets in routine clinical practice.^40^ Similarly, another study conducted in Ethiopia documented that more than half of patients had poor glycaemic control, with prevalence ranging from 52% to 68%, depending on clinical and behavioural factors.^41^ In addition, a recent systematic review and meta-analysis further confirmed that poor glycaemic control remains highly prevalent across Africa, often exceeding 60% of patients with type 2 diabetes.^42^ Comparable findings have also been reported in other studies, where poor glycaemic control was associated with long disease duration, poor medication adherence, obesity and inadequate self-care practices.^43^ These similarities may be explained by late diagnosis, insufficient patient education, limited access to HbA1c monitoring and suboptimal diabetes self-management support, which are common in resource-constrained healthcare systems.

However, lower prevalence rates have been reported in settings with stronger diabetes care systems where structured patient education, regular monitoring and multidisciplinary management are routinely implemented, indicating that health system strength plays a critical role in glycaemic outcome.^44^

Dyslipidaemia was present in 65.0% of the participants, indicating a substantial burden of lipid abnormalities among adults with T2DM. This finding is higher than the pooled prevalence estimates reported in a recent African systematic review and meta-analysis, which found prevalence rates of 52.7% for elevated low-density lipoprotein cholesterol, 43.5% for low-density lipoprotein cholesterol and 37.4% for hypertriglyceridaemia among individuals with type 2 diabetes. The higher prevalence observed in the present study may be attributed to poor glycaemic control, differences in lifestyle practices, obesity and variations in dyslipidaemia diagnostic criteria. However, the finding is consistent with reports from several hospital-based studies in sub-Saharan Africa that documented dyslipidaemia remains a major cardiometabolic risk factor and contributes significantly to the development of atherosclerotic cardiovascular diseases in the population.^45^

Renal impairment was observed in 29.2% of participants, suggesting that nearly one-third of the study population had evidence of impaired kidney function. This finding is comparable to reports from studies of adults with type 2 diabetes in which the prevalence of chronic kidney diseases or reduced eGFR ranged from 25% to 35%. Similar prevalence estimates of approximately 30% – 32% have been reported among individuals with type 2 diabetes, reflecting the substantial burden of diabetic kidney diseases globally. Conversely, lower prevalence rates of less than 20% have been documented in populations with earlier diabetes diagnosis, better glycaemic control and wider use of renoprotective therapies. The relatively high prevalence observed in the present study may be related to prolonged dyslipidaemia, coexisting hypertension, dyslipidaemia and delayed detection of kidney dysfunction. These findings underscore the importance of routine renal function assessment as part of comprehensive cardiometabolic risk evaluation in patients with type 2 diabetes.^37,46^

The mean cardiometabolic risk score of 1.87 ± 1.05 observed in this study indicates that, on average, participants had nearly two coexisting cardiometabolic risk factors, reflecting a substantial burden of metabolic and cardiovascular abnormalities among adults with T2DM. This finding supports the concept that cardiometabolic risk factors tend to cluster in individuals with diabetes, thereby amplifying the risk of cardiovascular diseases, chronic kidney diseases, and premature mortality. Similar studies have demonstrated that the accumulation of cardiometabolic abnormalities is strongly associated with adverse cardiovascular and metabolic outcomes, with risk increasing progressively as the number and severity of risk factors increase.^47,48^ The relatively high mean cardiometabolic risk score observed in the present study is consistent with the high prevalence of poor glycaemic control, dyslipidaemia, hypertension and renal impairment identified among the participants. These findings suggest that many patients were exposed to multiple interacting risk factors rather than isolated metabolic abnormalities. In contrast, studies conducted in populations with structured diabetes care, intensive risk-factor modification programmes and regular metabolic monitoring have reported a lower overall cardiometabolic risk burden, highlighting the importance of comprehensive management strategies.^49^ The findings underscore the need for routine cardiometabolic risk assessment and integrated management approaches that target multiple risk factors simultaneously to reduce the likelihood of cardiovascular and renal complications among individuals with T2DM.

The present study identified duration of diabetes, dyslipidaemia and renal impairment as significant predictors of poor glycaemic control among individuals with T2DM. Participants with a longer duration of diabetes were more likely to exhibit poor glycaemic control. These findings are consistent with previous studies that reported worsening glycaemic control with increasing disease duration because of progressive pancreatic β-cell dysfunction, declining insulin secretion and increasing insulin resistance. Chalie and colleagues reported that longer diabetes duration was significantly associated with delayed attainment of optimal glycaemic control among patients with type 2 diabetes.^41^ Similarly, Tegegne et al. identified longer disease duration as an important determinant of poor glycaemic outcomes in their systematic review and meta-analysis.^42^ Dyslipidaemia was also a significant predictor of poor glycaemic control in the present study. This association may be explained by the underlying insulin resistance that contributes to both hyperglycaemia and lipid abnormalities. Poor glycaemic control promotes increased hepatic lipoprotein production and impaired lipid metabolism, resulting in elevated triglycerides and increased low-density lipoprotein cholesterol. Similar findings have been reported in studies from Ethiopia and other sub-Saharan African countries, where dyslipidaemia was significantly associated with inadequate glycaemic control among patients with type 2 diabetes.^37,40^ Renal impairment emerged as another significant predictor of poor glycaemic control. Chronic hyperglycaemia contributes to progressive renal damage through glomerular hyperfiltration, oxidative stress and microvascular injury. Conversely, declining renal function may complicate glycaemic management through altered insulin metabolism and increased treatment complexity. Recent studies have demonstrated that patients with diabetic kidney disease are more likely to experience poor glycaemic control compared with those with preserved renal function.^37,46^ The association observed in this study therefore underscores the close interrelation between glycaemic status and renal dysfunction in individuals with type 2 diabetes. These findings highlight the importance of comprehensive cardiometabolic risk assessment and suggest that patients with longer diabetes duration, dsylipidaemia and renal impairment require closer monitoring and intensified management to achieve optimal glycaemic control and reduce the risk of diabetes-related complications.

Primary health care provides an effective platform for early detection and continuous monitoring of cardiometabolic abnormalities. Family-oriented care is particularly important because lifestyle modification, medication adherence and long-term disease management are influenced by family and household support systems. Community-based diabetes care approaches can improve patient education, promote regular screening, facilitate early detection of disease, and reduce the burden of diabetes-related morbidity in resource-limited settings. Strengthening community engagement is an essential strategy for improving diabetes outcomes.

The findings highlight the need to strengthen integrated diabetes management in PHC settings. Routine lipid profiling and renal function screening should be incorporated into chronic disease care pathways to facilitate early detection and prevention of cardiometabolic complications.

### Strength of the study

The study provides PHC-level evidence on the burden and clustering of cardiometabolic abnormalities among type 2 diabetes in North-Central Nigeria. By combining descriptive and analytical approaches, the study not only quantified prevalence but also identified significant associations and predictors. The use of standardised laboratory measurements and guidelines-based definition enhances the reliability and clinical relevance of the findings.

### Limitation of the study

The study is limited by its analytical cross-sectional design, which precludes causal inference and determination of temporal relationships. The relatively modest sample size may have reduced statistical power. In addition, the study was conducted in selected PHC clinics, which may limit generalisation of the findings. Although multivariable analyses were performed to adjust for potential confounders, residual confounding due to unmeasured or inadequately measured variables such as dietary patterns, medication adherence, physical activity and socioeconomic factors cannot be excluded. Therefore, the observed association should be interpreted with caution.

## Conclusion

The study demonstrated a high burden of cardiometabolic abnormalities among adults with type 2 diabetes in PHC clinics in Makurdi, Nigeria. Poor glycaemic control was associated with dyslipidaemia and renal impairment. Context-specific evidence from PHC settings is therefore essential to inform service integration, laboratory strengthening and chronic disease policy implementation.

### Recommendation

Healthcare workers should ensure comprehensive management of type 2 diabetes by routinely screening for cardiometabolic risk factors such as hypertension, dyslipidaemia and impaired renal function in addition to blood glucose monitoring. They should also provide continuous patient education on lifestyle modification, adherence to treatment and regular follow-up to reduce cardiovascular and renal complications.
